# Supplementary material for: 14,15-EET Reduced Brain Injury from Cerebral Ischemia and Reperfusion via Suppressing Neuronal Parthanatos
Source: Int J Mol Sci. 2021 Sep 7;22(18):9660. doi: 10.3390/ijms22189660 (PMC8471287; doi:10.3390/ijms22189660)
Supplement: Supplementary file 1 [file ijms-22-09660-s001.zip › ijms-1314719-supplementary.pdf]

supplementarySupplementary Figures

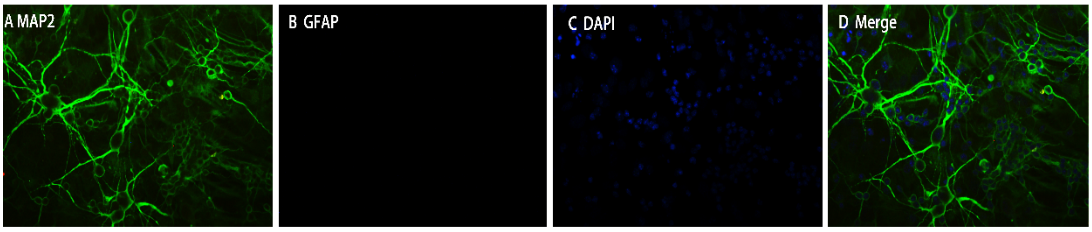

Supplementary Figure 1. Identification of neurons and astrocyte in primary culture cortical neurons at *DIV* 7. The cell were observed by immunofluorescence with MAP2 and GFAP antibodies. (A) Representative MAP2 positive cortical neurons (Green). (B) GFAP positive cell (Red). (C) Nuclues stained with DAPI (Blue). (D) Merge of A, B and C.
